# Automatic kidney segmentation using 2.5D ResUNet and 2.5D DenseUNet for malignant potential analysis in complex renal cyst based on CT images

**DOI:** 10.1186/s13640-022-00581-x

**Published:** 2022-03-22

**Authors:** Parin Kittipongdaja, Thitirat Siriborvornratanakul

**Affiliations:** Graduate School of Applied Statistics, National Institute of Development Administration, Bangkok, Thailand

**Keywords:** Kidney segmentation, Computed tomography, Deep learning, 2.5D convolution, ResUNet, DenseUNet

## Abstract

Bosniak renal cyst classification has been widely used in determining the complexity of a renal cyst. However, it turns out that about half of patients undergoing surgery for Bosniak category III, take surgical risks that reward them with no clinical benefit at all. This is because their pathological results reveal that the cysts are actually benign not malignant. This problem inspires us to use recently popular deep learning techniques and study alternative analytics methods for precise binary classification (benign or malignant tumor) on Computerized Tomography (CT) images. To achieve our goal, two consecutive steps are required–segmenting kidney organs or lesions from CT images then classifying the segmented kidneys. In this paper, we propose a study of kidney segmentation using 2.5D ResUNet and 2.5D DenseUNet for efficiently extracting intra-slice and inter-slice features. Our models are trained and validated on the public data set from Kidney Tumor Segmentation (KiTS19) challenge in two different training environments. As a result, all experimental models achieve high mean kidney Dice scores of at least 95% on the KiTS19 validation set consisting of 60 patients. Apart from the KiTS19 data set, we also conduct separate experiments on abdomen CT images of four Thai patients. Based on the four Thai patients, our experimental models show a drop in performance, where the best mean kidney Dice score is 87.60%.

## Introduction

Renal cysts (a.k.a., kidney cysts) are sacs of fluid or round pouches of fluid in kidneys. They can be classified as “simple” which are benign cysts (non-cancerous) or “complex” which might be malignant cysts (cancerous). The risk of malignancy can be quantified using Bosniak classification (developed in 1986 by Dr.Morton Bosniak), where CT scan is used to distinguish four renal cyst categories from I to IV, ranging from simple to complex cysts. This classification helps radiologists to determine each renal cyst as “nonsurgical” (i.e., benign in Bosniak categories I and II) or “surgical” (i.e., surgery is required in Bosniak categories III and IV). So far, this classification standard has remained the most commonly used classification to characterize renal cysts. However, in practice, Bosniak classification is subjective, meaning that poor extensive variations exist across different interpreters. According to the systematic review of [1], approximately 49% and 11% of those diagnosed in Bosniak categories III and IV, respectively, are found that their biopsy results indicate benign tumors (non-cancerous). Hence, their surgery operations are considered overtreatment and patients take risk of surgery without clinical benefit [2].

Recently, Machine Learning (ML) and Artificial Intelligence (AI) have been improved dramatically, particularly in Deep Learning (DL) for image understanding. In the past, it was necessary to manually design a set of visual features (a.k.a., handcrafted features) to develop AI-based medical imaging systems. However, in 2012, AlexNet [3], a deep learning-based Convolutional Neural Network (CNN) won ImageNet Large Scale Visual Recognition Challenge (ILSVRC) [4] with image classification results that totally outperformed other competitors both in the 2012 challenge and in previous challenges. Since the success of AlexNet in 2012, applications of deep learning in medical image analysis have gradually shifted to deep learning architectures, as these architectures share unique abilities to learn and generate useful visual features just by looking at image examples. In 2015, Microsoft’s ResNet [5] yielded a new low of 3.6% error rate in the history of ILSVRCs; this was the first time that computers surpassed the human-level performance of 5.1% in the ILSVRC image classification task. Consequently, the number of papers related to medical image analysis using deep learning techniques increased dramatically, particularly in 2015 and 2016 according to the survey in [6]. Apart from this, the survey in [6] concludes many interesting facts of medical image analysis. For example, segmentation was a task with the maximum number of medical imaging research papers; Magnetic Resonance Imaging (MRI), microscopy and CT were three medical imaging data types with top three numbers of research papers; there was a small amount of medical imaging research papers on abdominal CT image data. Besides, another recent paper of [7] reviews that the number of publications related to deep learning was exponentially increased for ‘deep learning and medical’ and ‘3D deep learning and medical’ keywords after the years 2015 and 2017 onwards, respectively.

The unnecessary surgery for Bosniak category III and the obvious trend of deep learning in medical imaging, inspire us to develop a deep learning-based analytical system that is capable of solving or alleviating the problem of Bosniak renal cyst classification (category III). Based on common practices found in current deep learning communities, we decide to split our task into two consecutive sub-tasks, i.e., kidney segmentation and Bosniak renal cyst classification, where each sub-task involves one deep learning model.

In the remaining of this paper, Sect. 2 reviews previous works. Section 3.1 walks through our first-hand experiences of preparing CT image data retrieved from one of the busiest public hospitals in Bangkok, Thailand. Section 3.2 explains our proposed deep learning models for kidney segmentation. Then, the models’ performances are evaluated and discussed in Sect. 4. Finally, Sect. 5 concludes this research paper. Note that, although this paper is written in the context of having Bosniak renal cyst classification as the final goal, the proposed models only deal with the first task of a deep learning model for kidney segmentation and there is no model proposed for Bosniak renal cyst classification yet.

## Related works

According to some previous works of deep learning-based kidney classification [8–10], one conclusion we found is that researchers tend to segment only the kidney mass from CT images before proceeding to the classification step.

So far, kidney segmentation (a.k.a., renal segmentation) has remained a challenging task for CT images. This is because kidney mass can be found in abdominal CT images where not only the kidney but other organs such as liver, pancreas, spleen, and GI tract are residing in. The comprehensive survey in [11] shows that, over the past decades, numerous traditional segmentation techniques for CT images have been proposed, including manual segmentation, thresholding segmentation, region-based segmentation, model-based segmentation, atlas-based segmentation, graph-based segmentation, and hybrid segmentation. However, these traditional techniques have limitations in the context of kidney segmentation regarding CT images. For example, simple techniques such as thresholding segmentation and model-based segmentation are sensitive to noises and unable to deal with large intensity variations in abdominal CT images; atlas-based segmentation and graph-based segmentation require user initialization, which is not automatic and inter-rater variability can affect segmentation performance.

In recent years, deep learning techniques with CNN have achieved superior performance in numerous fields of computer vision, including semantic segmentation, image classification, object detection, and so on. For salient image segmentation tasks, it can be said that deep learning is now one of the most popular techniques. For example, in 2016, Zhou et al. [12] propose a fully convolutional neural network (FCN) for semantic segmentation on CT images. In this work, VGG-16 is used as the backbone CNN architecture. Using all three anatomical planes, this work utilizes CT image data from 230 patients (84,823 image slices) for training and 10 patients for testing. According to their evaluation, the results in terms of the mean value of Intersection Over Union (IoU) or Jaccard index for right and left kidneys are 0.86 and 0.84, respectively. Later, in 2017, Zhou et al. [13] improve their previous work by defining the range of target regions based on organ localizations; this helps increase IoU of the left kidney to 0.88. Note that in this improved work, the numbers of patient cases are changed to 228 patients for training and 12 patients for testing.

In 2017, Sharma et al. [14] present the automated segmentation of ADPKD (Autosomal Dominant Polycystic Kidney Disease) kidneys using an FCN trained end-to-end on slicewise axial-CT sections. This work uses VGG-16 as a backbone CNN, but only axial plane images are considered. Their data consist of 165 patients (16,000 original CT image slices which are increased to 48,000 image slices after data augmentation) for training and 79 patients (9000 CT image slices) for testing. Their proposed method yields the mean Dice similarity coefficient (DSC) of 0.86 ± 0.07 (mean ± SD) when comparing this automated segmentation with the ground-truth manual segmentation (done by clinical experts). However, if a patient has liver cysts in close proximity to the kidney, this automated segmentation tends to overestimate the kidney volume due to the inclusion of liver cysts in the segmented kidney region. Also, in rare cases of kidneys with extremely high total volume (>13,000 mL), this proposed method cannot exploit context information around the kidney, leading to poor segmentation results.

In 2018, Jackson et al. [15] employ an automated CNN-based software tool to perform quantitative analysis of SPECT images based on the anatomical outline in a fused CT volume. U-Net architecture is used on axial-plane CT image slices. Their data set includes 89 patient cases for training and 24 patient cases for testing. The proposed model achieves mean Dice scores of 0.91 and 0.86 for right and left kidneys, respectively.

Although many previous studies achieve good segmentation using deep learning techniques, to the best of our knowledge, we have found no study mentioned generalizability either to a different data source or to a different quality of images. Fundamentally, image quality in CT, as in all medical imaging, depends on four primary factors [16]—image contrast, spatial resolution, image noise, and artifacts. Moreover, CT images are typically stored in Digital Imaging and Communications in Medicine (DICOM) file format and the different environments of DICOM files could affect segmentation performance as mentioned in previous studies [12–15].

## Methods

### Data preparation

#### Informed consent

This research is a retrospective cross-sectional study of 200 patients who had taken abdominal CT images from January 1, 2007 to December 31, 2019. The CT image data of Thai patients are provided by the Department of Radiology and Department of Pathology, Faculty of Medicine, Ramathibodi Hospital, Bangkok, Thailand. To retrieve abdominal CT images from the hospital’s database, we follow the principles of Helsinki Declaration and under Medical Research Involving Human Subjects Act. The protocol of this research is approved by the ethics committee of Ramathibodi hospital. While conducting this research, patients’ information is protected using encrypted Hospital Number (HN) with the MD5 hash function known only by the principal investigator. The encrypted HN is used during feature extraction and data analysis processes with no disclosure of patient information at all. Note that the need for individual informed consent is waived by the ethics committee of Ramathibodi hospital, Bangkok, Thailand.

#### Data retrieval, selection and annotation

Data retrieved from Ramathibodi hospital’s database consists of two parts. The first part is abdominal CT images in all three anatomical planes (i.e., axial, coronal, and sagittal planes) and in all phases of contrast media injection. These CT images are stored in DICOM file format with accompanying details, such as resolution, slice thickness, HN, etc. The second part of retrieved data are pathological test results that confirm malignancy of renal cysts together with HNs that link back to corresponding abdominal CT images.

In this phase of data preparation, many problems arose. First, it took us 5–6 months for data request and protocol approval, as there was an unexpected delay due to COVID-19 outbreak. Second, the pathological test results were stored in a way that did not allow any automatic or programming-based retrieval; the only way to get them was to connect to the hospital’s intranet, access each patient’s Portable Document Format (PDF) radiology report, and read the report manually. Third, according to the actual pathological test results, the number of patients with Bosniak category III and IV was way too small-only 19 patient cases from the total of 200 cases. This unexpectation was crucial as data from 19 patient cases was not enough for training complicated deep learning models regarding kidney mass segmentation. To get around with this insufficient number of patient cases, in this research, we started training and validating our kidney segmentation models (Sect. 3.2) with a public data set of 300 patients from the official 2019 KiTS challenge [17] (the data set was available for download at https://github.com/neheller/kits19). Then, we tested the trained models on our CT images of Thai patients, examining how the domain shift affected segmentation performance.

The last problem we encountered is the classic problem of laborious data annotation tasks that consumed too much time from human physicians. Because CT images retrieved from Ramathibodi hospital came with no segmenting annotation, we had to ask an experienced urologist to help annotate data for us, despite her regular workload as a urologist in one of the busiest public hospitals in Bangkok, Thailand. The annotation was done with a python-based graphical image annotation tool named labelme 4.5.1 [18] and it took 1–5 min for the urologist to annotate one image slice. Hence, for one patient with 70 image slices, the annotation task cost 70–350 min from the urologist. After waiting for 3–4 weeks, we decided to continue developing our prototype models using data of four Thai patients (two with benign renal cysts and two with malignant renal cysts), whose 70 image slices (per one patient) were properly annotated.

In conclusion, our models are trained on the KiTS19 public data set [17] that consists of cross-sectional CT data from 300 patients with kidney tumors (210 patients for training and 90 patients for testing). However, as KiTS19’s testing data of 90 patients do not have accompanied ground truth images, we exclude these data from our supervised training. In a detail of the KiTS19 data set, each patient includes 400–600 CT image slices whose slice thickness ranging from 1–﻿5 mm, and each CT image slice has a 2D dimension of 512$\times$512 pixels. Although all patients (mostly American) in this data set were treated at the same University of Minnesota Health, more than half of the imaging was obtained from 50 referring institutions, making this data set a diverse data set in terms of CT scan settings. As for the CT data of Thai patients to be used in our domain shifting test, we choose only abdominal CT image slices in venous phase contrast media injection, as this phase provides the clearest visibility of kidneys. One Thai patient has about 100–200 image slices (the slice thickness ranges from 2–3 mm and one slice has a 2D resolution of 512$\times$512 pixels) per one phase of contrast media injection but only 70 image slices containing kidney mass are used in our experiments.

#### Data preprocessing

In this section, we describe all preprocessing steps regarding CT data stored in DICOM file format.

*Rescaling* this preprocess step refers to converting CT numbers as originally stored in pixels of CT image slices into Hounsfield Unit (HU) of radiodensity measurement. In CT scan, each pixel of 2D CT image slices is originally assigned to a numerical value called CT number-the average of all attenuation values contained within the corresponding voxel, where voxel is a volume element in the patient and voxel volume refers to the product of pixel area and slice thickness. This CT number can be converted to HU scale to reflect the electron density of the imaged tissue at a given location. Each HU value is then assigned to one grayscale intensity to form a digital image—the higher the HU value, the brighter the pixel intensity. The conversion from CT number to HU scale can be done with the following equation:

1 $$\begin{aligned} HU = (rescale\_slope * CT\_number) + rescale\_intercept \end{aligned}$$

where $rescale\_slope$ and $rescale\_intercept$ (as stored in DICOM file) are 1.0 and − 1024.0, respectively, for our Thai patient data. As for the KiTS19 data set, data are already in HU scale, so there is no need for us to do this conversion.

*Window Level (WL) and Window Width (WW)* due to the fact that there are 2000 shades of gray and might be as many as 4000 shades in modern CT scan devices, it is beyond human vision to distinguish such a wide range of grayscale shades. Likewise, DICOM files are high-resolution grayscale images (64-bit with Hounsfield unit) that might not be suitable with most deep learning models, whose default format is 32-bit floating-point. Hence, it is necessary to clip or cutoff only the HU range of interest.

In medical practice, shades of gray are scoped to a specific range of values to display tissues or organs of interest; any value outside the range is assigned to black. The parameters used to specify the range are called Window Level (WL) and Window Width (WW). WL is the value used to specify the middle of HU value to display as the main intensity. For example, for the bone tissue of range + 400 to + 1000 HU values, WL should be set to the middle of + 700 HU. WW is the value used to specify the width or range of grayscale level to be displayed. To cover all possible HU values regarding the organ of interest, WW has to be set in according to WL. For example, using WL = + 700 HU and WW = + 200 HU results in the display of range + 600 to + 800 HU which does not cover the entire range of bone tissue.

In this research, we focus on the renal organ whose HU values are in the range of + 20 to + 40 HU. Therefore, we set our range of HU to the minimum of − 73 and the maximum of + 304, ensuring coverage visibility of kidneys. After clipping HU values to our specified range, we normalize all HU values to the range of 0.0–1.0 to yield faster convergence according to the best practice in training deep learning models.

*Image Registration* this is the preprocessing step for geometrically aligning image slices taken at different times and angles. As for the data set of KiTS19 and our Thai patient data, there is no unalignment problem, so this preprocessing step is skipped.

*Data Augmentation* we employ data augmentation to improve variability and diversity of the training data in KiTS19. Our data augmentation is done by a library named albumentations 0.2.3 [19] (https://albumentations.ai/), including horizontal flip (*p* = 0.5), random brightness contrast (*p* = 0.5), random gamma (*p* = 0.5), and grid distortion (*p* = 0.5, border_mode=cv2.BORDER_ CONSTANT).

### Deep learning models for kidney segmentation

To segment kidney mass from one CT image slice, 2D image segmentation algorithms are appropriate solutions. However, when kidney mass has to be segmented continuously from a sequence of CT image slices, the problem becomes harder and requires more complicated solutions of 3D image segmentation for smooth segmentation over the sequence. Speaking of 3D image segmentation in the medical domain, many successful algorithms are based on 3D variants of the U-Net architecture [20]. In addition, many works have recently suggested that the deeper the network, the better the performance [21]. Likewise, according to Heller et al. [22], deep 3D CNNs are said to be the most popular methods so far in KiTS19 Challenge’s submissions.

In practice, it is challenging to train a very deep neural network from scratch due to problems, such as vanishing gradients. To overcome this problem, the concept of skip connections (a.k.a., residual connections) introduced in Microsoft’s ResNet [5] comes in handy. For example, Zhang et al. [23] propose a deep ResUNet network for road segmentation, where ResUNet refers to a semantic segmentation neural network that combines the strengths of U-Net and residual neural network. This combination brings two main benefits: (1) the residual unit helps ease the training as some layers in the network can be skipped and (2) the skip connections help facilitate information propagation with no degradation, making it possible to design a neural network with much fewer parameters but with comparable or even better performance on the task of semantic segmentation. Hence, in this research paper, we decide to use the concept of deep ResUNet to compromise between the network’s depth and the training’s practicality.

As for the question of which alternative should we use between 2D and 3D convolutions. It is obvious that 2D convolution which processes a volume slice-by-slice cannot fully leverage the spatial information along the third dimension. However, 3D convolution as preferred by many previous works of 3D medical image segmentation suffers from high computational cost and significant Graphics Processing Unit (GPU) memory consumption. To compromise between accuracy and computational resource, this research paper goes in between and utilizes the concept of 2.5D convolution to incorporate partial 3D information. This is the same strategy used by other works of organ segmentation, for example, brain [24, 25], pancreas [26] and liver [27] segmentation.

**Fig. 1:**
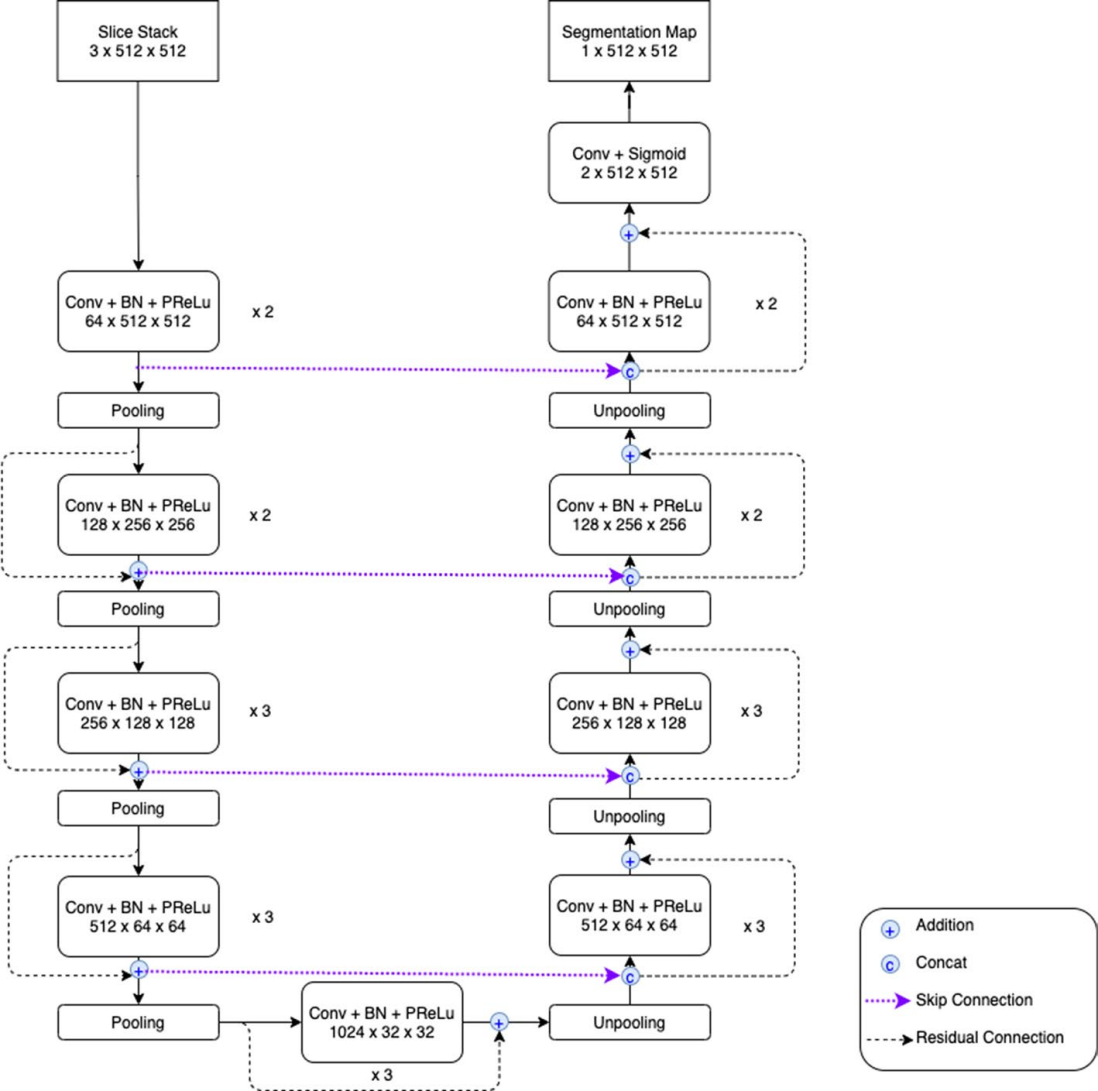
2.5D ResUNet architecture with the slice stack of *n* = 3. Please note that this diagram is created by the desktop software named diagrams.net (https://app.diagrams.net/), version 14.5.0

Inspired by the architecture proposed in Tsai and Sun [28], our experimental architecture of 2.5D convolution on ResUNet is illustrated in Fig. 1. At first glance, it looks very similar to ResUNet using normal 2D convolutions. However, the strategy of 2.5D convolution takes place at the very beginning of ResUNet which is illustrated as the box named “Slice Stack $n \times 512\times 512$” in Fig. 1. In this additional box, instead of using only one CT image slice input (1$\times$512$\times$512) to get one segmentation output (1$\times$512$\times$512), we use a stack of *n* adjacent CT image slices ($n\times 512\times 512$) as the input to ResUNet where the middle slice is our slice of interest where the segmentation result of ResUNet (1$\times$512$\times$512) will correspond to. This *n*-slice input provides larger image content in the axial plane and extra contextual information in the orthogonal direction, introducing partial 3D information into the model. Note that in our experimental model architecture, all convolutional layers share the same kernel size of 3$\times$3 and use a nonlinear activation function of Parametric Rectified Linear Unit (PReLU). Apart from faster computation, using 2.5D convolution instead of actual 3D convolution also has an advantage that allows us to increase the model’s complexity by adding more layers and using a much larger number of feature channels in each layer.

**Fig. 2:**
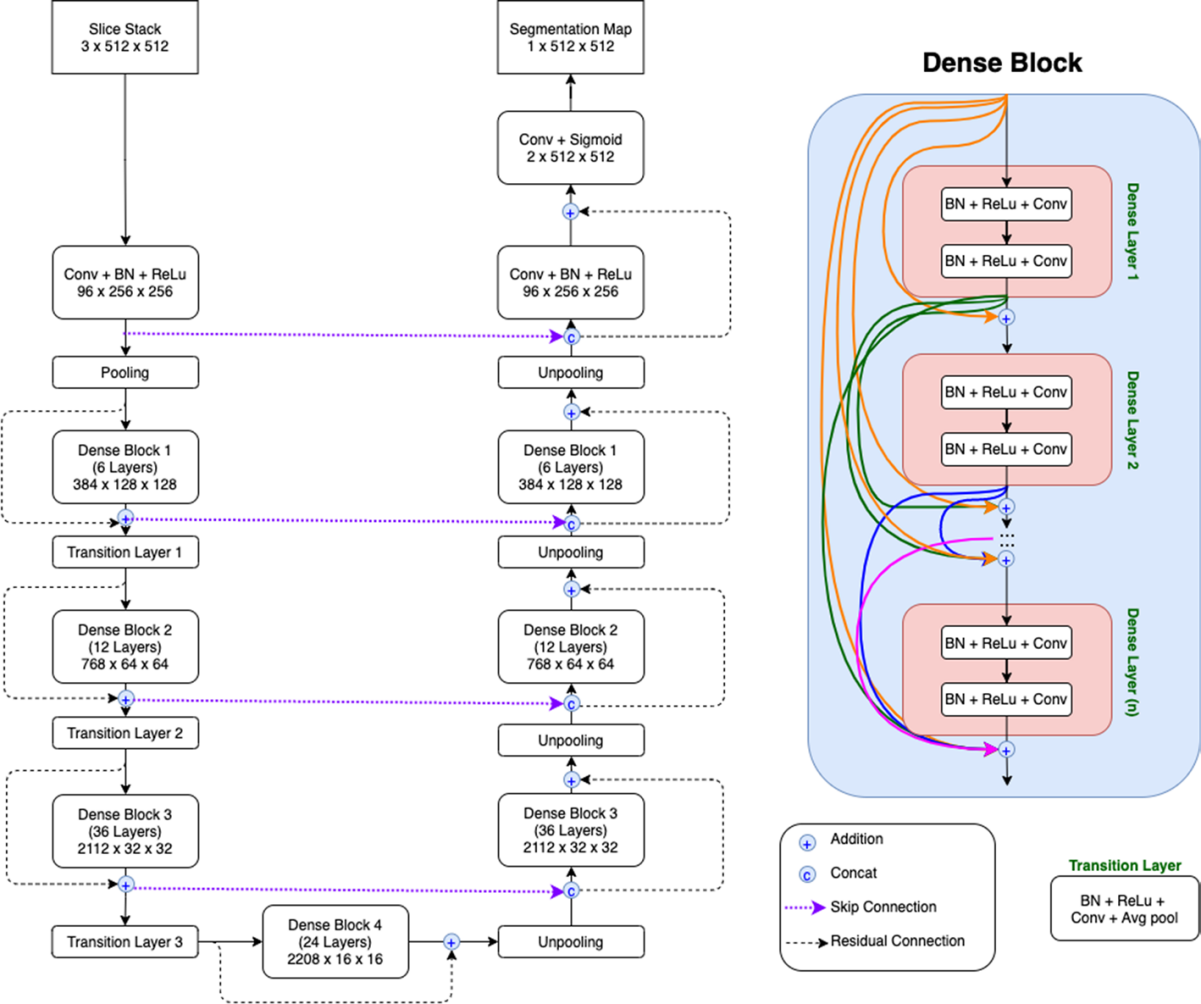
2.5D DenseUNet architecture with the slice stack of *n* = 3. Please note that this diagram is created by the desktop software named diagrams.net (https://app.diagrams.net/), version 14.5.0

To get more comparative results, we also conduct experiments on another deep learning architecture called DenseUNet [28]. Nowadays, most CNN-based approaches are implemented with seminal backbone networks, among them the two arguably most famous ones are ResNet [5] and DenseNet [29]. On one hand, the residual connections in ResNet help promote information propagation, both forwardly and backwardly, through the network. However, the identity shortcut that stabilizes training might limit its representation capacity. On the other hand, the connected path of DenseNet ensures the maximum information flow between layers, which improves the gradient flow, and thus alleviates the burden in searching for the optimal solution in a very deep neural network. As these two backbone networks have unique strengths, we decide to experiment with both and call them ResUNet and DenseUNet respectively. In this work, the architecture of DenseUNet is shown in Fig. 2; it is the extended version of 2.5D ResUNet that borrows the concept of dense connections from the famous DenseNet-161. As seen in Fig. 2, input feature maps of the shallower layers are used as inputs to all subsequent (deeper) layers in the same dense block as well. This concept of dense connections not only helps ease the problem of gradient vanishing but also allows reusing feature maps from the shallower layers in the deeper layers.

Our two experimental model architectures are implemented with PyTorch 1.5.0 + cu101 library based on Python 3.6 programming language. Using the KiTS19 data set, our models are trained and validated in two training environments: Google Colab Pro (NVIDIA Tesla P100-PCIE GPU with 16GB memory) and NVIDIA DGX A100 (NVIDIA A100 GPU with 40GB memory). Google Colab Pro is a cheap and accessible training alternative that is based on a cloud GPU whereas NVIDIA DGX A100 refers to a local non-cloud GPU. Due to the limited GPU memory of Google Colab Pro’s environment, only 2.5D ResUNet architecture gets trained for 30 epochs using the mini-batch size of 8, cross-entropy loss function, and Adam optimizer (initial learning rate is set to 0.0001 and multiplied by 0.1 when loss does not decrease for five consecutive epochs). Nevertheless, training our 2.5D ResUNet model on data of 210 patients in KiTS19 requires 30 h (approximately) per one epoch of training because of the slow reading speed between Google Colab and Google Drive. As Google Colab Pro has a limitation of 24-h continuous runtime, we have no choice but to reduce the training time per one epoch. Hence, from 210 patients of the KiTS19 data set, we choose the first 60 patients as our validation set and the last 60 patients as our training set. As a result, our 2.5D ResUNet model can be trained on Google Colab Pro using 9–10 h per one epoch.

In the local non-cloud training environment of NVIDIA DGX A100, not only that we have more available GPU memory but also there is no runtime limitation and no overhead time in reading data from different servers. As a result, we are able to train both experimental architectures with a bigger mini-batch size of 16, a training set of 150 patients, and a validation set of 60 patients. Other training parameters remain the same as described in the previous paragraph. In this non-cloud training environment, the 2.5D ResUNet architecture can be trained in just 20 min per epoch using 28GB GPU Video Random Access Memory (VRAM), and the 2.5D DenseUNet architecture can be trained using 30 min per epoch and 39GB GPU VRAM. Both architectures are trained for 20 epochs.

## Results and discussion

To evaluate the performances of our trained models regarding kidney segmentation, we use Dice coefficient (a.k.a., F1 score for segmentation) which is a measure of overlap between two samples. This measure has a floating-point value ranging from 0.0 to 1.0, where Dice coefficient of 1.0 denotes perfect and complete overlap. Dice coefficient is originally developed for binary data and can be calculated as

2 $$\begin{aligned} DICE = \frac{2|A \cap B|}{2|A|+|B|} \end{aligned}$$

where $|A \cap B|$ represents the common elements between A and B, and |*A*| represents the number of elements in A (and likewise for |*B*|). As for our task of evaluating a predicted segmentation mask against a ground-truth segmentation mask, we can approximate the term $|A \cap B|$ as the elementwise multiplication between the prediction mask and the ground-truth mask, then sum the resulting matrix into a final result of one scalar value. Because values in our ground-truth mask are binary of either 0 or 1 whereas values in the predicted mask are floating-point of range 0.0–1.0, we can effectively zero-out any pixels (in the predicted mask) which are not activated in the ground-truth mask. Finally, Dice score for one patient with many CT image slices is computed by averaging Dice scores from all image slices. Note that the same averaging computation is applied to compute Dice scores regarding train/validation/test sets.

Our experimental results are concluded in Table 1, where the best performance on Thai patients is from 2.5D DenseUNet using slice stack of 5. When evaluating on 150 patient data from the KiTS19’s train set (the second column), our five experiments show comparable Dice scores compared to the two 3D U-Net models as reported in [30]. Nevertheless, our Dice scores in all experiments slightly drop when being used with unseen 60 patient data in the KiTS19’s validation set (the third column), and significantly drop when being used with Thai patients (the fourth column). This conveys a classic problem of performance drop due to domain shifting. Among our five experiments, the worst performance on Thai patients is the one trained in Google Colab Pro’s environment. Our assumption is that limited computational resources and runtime constraints in Google Colab Pro’s environment not only introduce inflexibility in experiments but also hurt the performance of deep learning models as bigger models, larger batch sizes and more training data are not possible in this training environment.

**Fig. 3:**
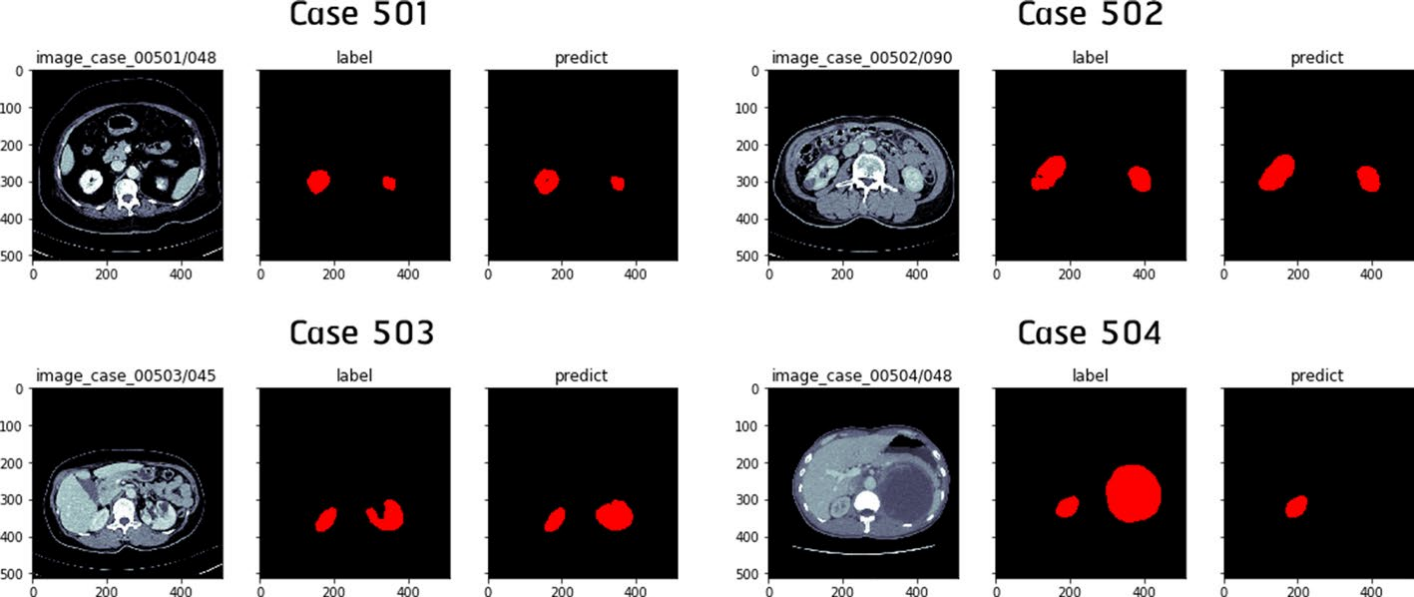
Kidney segmentation results regarding four Thai patients using 2.5D ResUNet (slice stack of 5) trained in Google Colab Pro environment

**Fig. 4:**
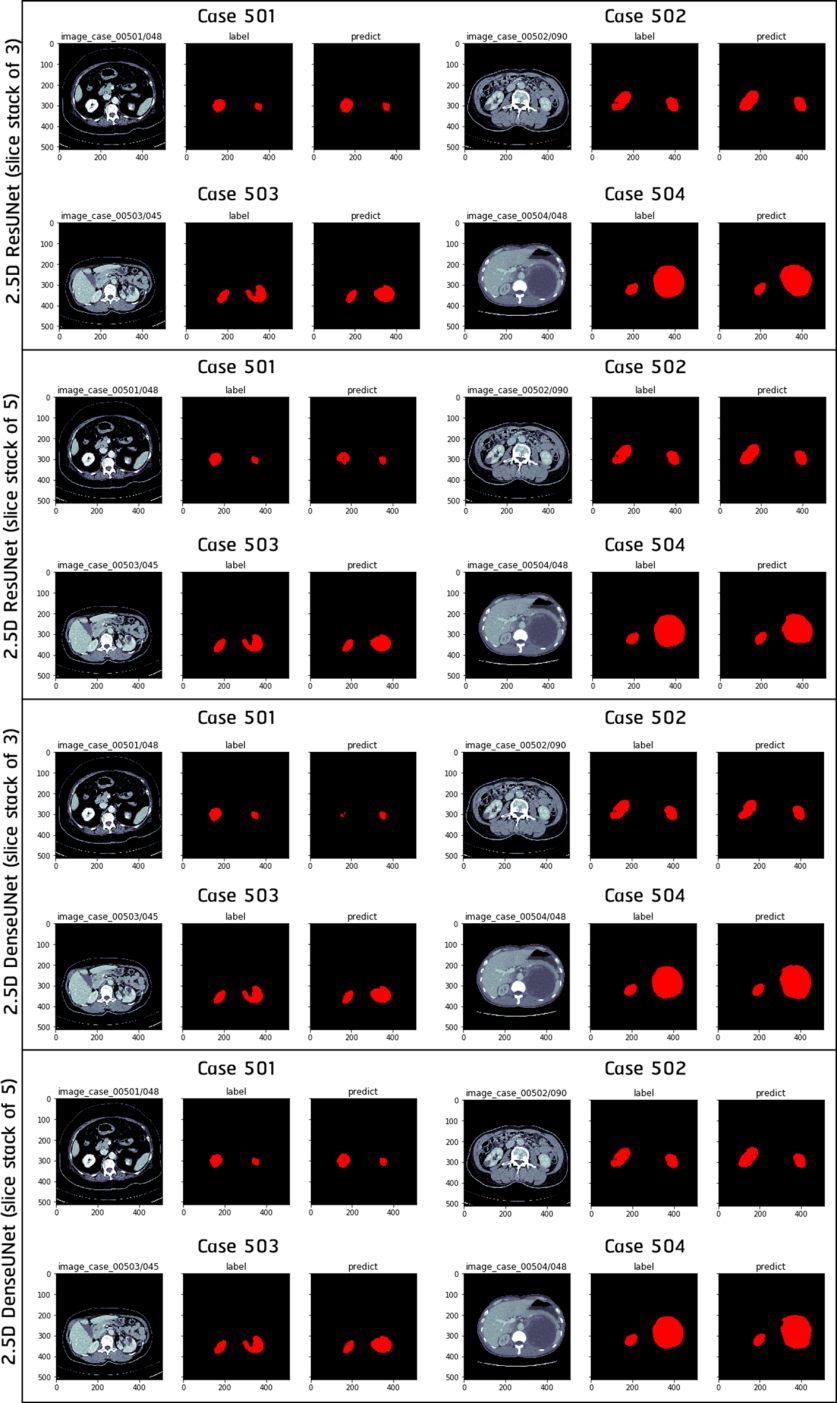
Kidney segmentation results regarding four Thai patients using models trained in NVIDIA DGX A100 environment. From top to bottom are 2.5D ResUNet (slice stack of 3), 2.5D ResUNet (slice stack of 5), 2.5D DenseUNet (slice stack of 3), and 2.5D DenseUNet (slice stack of 5)

Table 2 shows per-patient Dice scores regarding three experiments sharing the same slice stack of 5. From this table as well as from Figs. 3 and 4 , one clear conclusion is that segmentation performances tend to drop in Thai patients with malignant renal cysts (patient 502 and 504). Besides, it can be seen that 2.5D DenseUNet always yields higher Dice scores than 2.5D ResUNet.

In conclusion, there are two main limitations in our current work. First, because the testing size of Thai patients was very limited, the model accuracy computed from this small test set might not be a good statistical representation of Thai patients. Second, our current work suffers from the problem of poor generalization due to data distribution shifts. Early work [31] has shown a similar problem that CT data sets on classification models may not generalize well when externally validated on data from a different institution. This is because the CT data sets are possibly vulnerable to distribution shifts stemming from changes in patient population or rely on non-medical relevant cues between institutions. Therefore, the presented results in this paper might not represent the true performance of the network to Thai patient kidney segmentation.

**Table 1:** Experimental results regarding the KiTS19 data set and our four Thai patients

**Model architecture**	**Averaged Dice score**		
	**KiTS train**	**KiTS val**	**Thai patients**
Google Colab Pro:			
2.5D ResUNet (slice stack of 5)	**0.9801**	0.9373	0.6900
NVIDIA DGX A100:			
2.5D ResUNet (slice stack of 3)	0.9735	0.9554	0.7977
2.5D ResUNet (slice stack of 5)	0.9772	0.9567	0.7335
2.5D DenseUNet (slice stack of 3)	0.9779	**0.9595**	0.8367
2.5D DenseUNet (slice stack of 5)	0.9769	0.9582	**0.8760**
Comparative results from [30]:			
3D U-Net	0.9734	-	-
Residual 3D U-Net	0.9736	-	-

The best result regarding each column is highlighted in bold

**Table 2:** Detail experimental results regarding each Thai patient

Model architecture	Dice score per one patient				Averaged dice score
	501 (benign)	502 (cancer)	503 (benign)	504 (cancer)	
Google Colab Pro:					
2.5D ResUNet (slice stack of 5)	0.8650	0.6591	0.9128	0.3231	0.6900
NVIDIA DGX A100:					
2.5D ResUNet (slice stack of 5)	0.7608	0.4480	0.9050	0.8201	0.7335
2.5D DenseUNet (slice stack of 5)	**0.9014**	**0.7546**	**0.9132**	**0.9347**	**0.8760**

The best result regarding each Thai patient is highlighted in bold

## Conclusion and future works

In this paper, we propose our study and experiments of using deep learning techniques towards the long-term goal of easing the problem of unnecessary surgery for patients with Bosniak category III and IV diagnoses. The current research focuses on the very first step towards Bosniak renal cyst classification-deep learning models for segmenting kidney mass from high-dimensional CT data. To compromise computational resources and accuracy, our experimental architectures are based on two combined concepts of 2.5D convolution and skip connections. Two architectures are experimented (i.e., 2.5D ResUNet and 2.5D DenseUNet) in two different training environments. The experimental architectures are trained and validated on the KiTS19 public data set then tested for domain shifting on our CT data of Thai patients. Experimental results suggest that training deep learning models in low computational resources can significantly hurt the performance. In addition, our experimental results show superiority of DenseUNet over ResUNet and there are segmentation accuracy drops regarding domain shifting and regarding Thai patients with malignant renal cysts.

To draw a more concrete conclusion regarding Thai patients, more annotated data from Thai patients is required. As future works, first, we plan to experiment on transfer learning and fine-tuning, investigating whether it is possible to simply transfer knowledge learned from KiTS19 to our data set in a kidney segmentation task. If the first experiment cannot yield our desired performance, we are interested in using another deep learning technique to learn the conversion between latent variables of the two data sets, so that images from these two different domains can be converted to match each other. Other interesting alternatives are to explore recently advanced techniques such as a multi-view deep neural network [32] to incorporate image slices from other scanning axes into consideration, no-reference image quality assessment [33] for better image quality evaluation, and image denoising [34] for enhancing the quality of each image slice.

## Data Availability

The KiTS19 data set used in this paper can be found at https://github.com/neheller/kits19. As for the data of Thai patients, we are not able to share them publicly as this is prohibited by the ethics committee of Ramathibodi hospital, Bangkok, Thailand.

## Abbreviations

AI: Artificial intelligence
CNN: Convolutional neural network
CT: Computerized tomography
DICOM: Digital imaging and communications in medicine
DL: Deep learning
GPU: Graphics processing unit
HN: Hospital number
HU: Hounsfield unit
ILSVRC: ImageNet large scale visual recognition challenge
IoU: Intersection over union
KiTS19: Kidney tumor segmentation
ML: Machine learning
MRI: Magnetic resonance imaging
PDF: Portable document format
PReLU: Parametric-rectified linear unit
VRAM: Video random access memory
WL: Window level
WW: Window width
